# A programmable metasurface with dynamic polarization, scattering and focusing control

**DOI:** 10.1038/srep35692

**Published:** 2016-10-24

**Authors:** Huanhuan Yang, Xiangyu Cao, Fan Yang, Jun Gao, Shenheng Xu, Maokun Li, Xibi Chen, Yi Zhao, Yuejun Zheng, Sijia Li

**Affiliations:** 1 Information and Navigation Institute, Air Force Engineering University, Xi’an, 710077, China; 2 Department of Electronic Engineering, Tsinghua University, Beijing, 100084, China

## Abstract

Diverse electromagnetic (EM) responses of a programmable metasurface with a relatively large scale have been investigated, where multiple functionalities are obtained on the same surface. The unit cell in the metasurface is integrated with one PIN diode, and thus a binary coded phase is realized for a single polarization. Exploiting this anisotropic characteristic, reconfigurable polarization conversion is presented first. Then the dynamic scattering performance for two kinds of sources, i.e. a plane wave and a point source, is carefully elaborated. To tailor the scattering properties, genetic algorithm, normally based on binary coding, is coupled with the scattering pattern analysis to optimize the coding matrix. Besides, inverse fast Fourier transform (IFFT) technique is also introduced to expedite the optimization process of a large metasurface. Since the coding control of each unit cell allows a local and direct modulation of EM wave, various EM phenomena including anomalous reflection, diffusion, beam steering and beam forming are successfully demonstrated by both simulations and experiments. It is worthwhile to point out that a real-time switch among these functionalities is also achieved by using a field-programmable gate array (FPGA). All the results suggest that the proposed programmable metasurface has great potentials for future applications.

Metamaterials, normally composed by artificially periodic or quasi-periodic structures with sub-wavelength scales, provide a new design strategy for functional materials^1^,^2^. In the last decades, unusual electromagnetic (EM) properties of metamaterials such as negative permittivity and permeability^3^,^4^, which are unavailable in natural materials, have been presented. Numerous extraordinary EM phenomena have been observed in microwave^5^, terahertz^6^ and optical fields^7^, and various novel devices like invisibility cloak^5^,^8^, lens^9^,^10^ and perfect absorber^11^,^12^ have been demonstrated. Evolved from bulky three-dimensional metamaterials, a metasurface, as a two-dimensional equivalent of metamaterial, has an ultrathin profile that considerably reduces the volume and weight, and simplifies the fabrication and integration process. Consequently, it exhibits tremendous application potentials and has attracted great interests in recent years^13^,^14^,^15^,^16^,^17^,^18^. Initially, the combinations of artificially periodic metallic or dielectric structures with natural homogeneous material have been utilized to construct metasurfaces. The control of magnitude^11^, phase^19^ or polarization^20^ of EM wave has been successfully realized. Subsequently, it is found that metasurfaces with quasi-periodic or aperiodic structures provide greater design freedom and thus are able to produce more flexible manipulation of EM wave. More powerful functions such as broadband diffusion^17^,^21^, anomalous refractions^16^,^22^ and reflections^7^,^23^ have been achieved, resulting in many novel functional devices including mantle cloak^13^,^24^,^25^, flat metalens^26^,^27^ and Huygens’ surfaces^28^, etc.

Most of aforementioned metasurfaces have focused on a certain function, so that the manipulation of EM wave is fixed once the design is completed. Recently, much attention has been paid on tunable metasurfaces whose operation status can be dynamically controlled, and hence flexible functionalities are expected. Several excellent work has been reported to demonstrate the steerable manipulation of the transmitted or reflected EM wave. By incorporating active components into each unit cell in the metasurface, the electronic tuning of the fundamental properties of EM wave has been successfully attained, such as the tunable absorbance^29^, the full 360° reflection phase tuning^30^, the separate control of amplitude and phase^31^, and the polarization manipulation^18^, etc. Although these metasurfaces have great potentials in versatile EM wave manipulations, more advanced functionalities have not been shown due to the identical electronic control of all the unit cells in a metasurface. Quite recently, a few pioneer metasurfaces have also been reported to illustrate the real-time realization of different functions. In ref. 32, the electronically multi-beam scattering has been presented. Moreover, the concept of coding metasurface has also been proposed, providing a powerful tool in functional metasurface design. In ref. 33, a field-programmable array antenna has been realized using a reflective coding metasurface and beam steering performance is obtained. In ref. 34, the beam forming functionality of a transmission-type programmable metasurface has been applied in microwave imaging. While a variety of functionalities have been successfully presented with different metasurfaces, the simultaneous demonstration of different functionalities for one metasurface has not been fully reported. Furthermore, there is little study on the reconfigurable manipulation of polarization. Especially with the increasingly diversified demands nowadays, it is believed that multifunctional metasurfaces have more application potentials.

In this article, we present the dynamic multi-functional properties of a digitally controlled metasurface with a relatively large aperture size (>20 wavelengths). The proposed metasurface is constructed by jointing 5 identical sub-metasurfaces, and each sub-metasurface consists of 320 active unit cells. By integrating one PIN diode into each unit cell, a reconfigurable phase is realized for a single polarization. Utilizing this anisotropic property, the reconfigurable polarization conversion is realized first. A comprehensive display of various functionalities including agile scattering, planar focusing, beam steering as well as beam forming is also presented by programming a coding matrix with the aid of genetic algorithm (GA) and inverse fast Fourier transform (IFFT) technique. Furthermore, the real-time switch among these functions is achieved by using a field-programmable gate array (FPGA). Compared with the previous work normally controlling a lattice and only focusing on one type of steerable functions, each unit cell in the proposed metasurface can be controlled independently, and thus more versatile functions are achievable simultaneously with our design. The results clearly show that the proposed programmable metasurface can be applied to a variety of applications, including smart stealth missions and novel phased array technique, without the need of expensive phase-shifting components.

## Results

### Coding unit cell, programmable metasurface, and coding optimization

**Figure 1:**
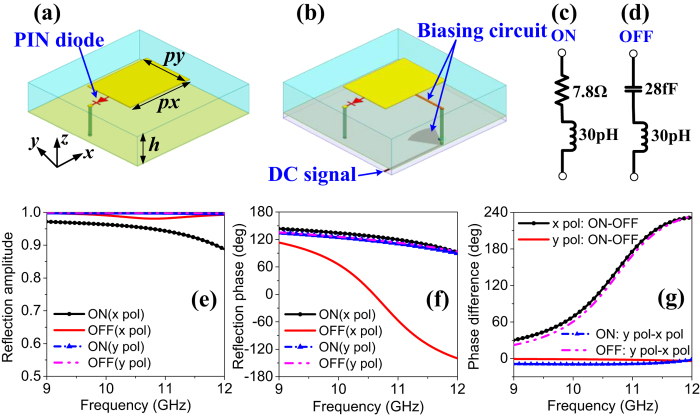
Proposed unit cell and its coding property. (**a**) Schematic of proposed unit cell integrated with one PIN diode. The detailed dimensions are *px* = 5.8 mm, *py* = 4.9 mm, *h* = 1.58 mm. (**b**) Proposed unit cell with actual biasing architecture. The biasing circuit is elaborately designed to isolate the direct-current (DC) and radio frequency (RF) signals. (**c,d**) The equivalent circuits of the PIN diode (MACOM MADP-000907-14020) at ON and OFF states, respectively. (**e**) Reflection amplitudes. (**f**) Reflection phases. (**g**) Reflection phase differences.

The schematic of the proposed unit cell for multi-functional metasurface is presented in Fig. 1(a). It has a sandwich structure composed of a simple rectangular patch and a metal-ground plane spaced by a substrate of Taconic TLX-8 whose dielectric constant is 2.55 and loss tangent is 0.0019. A PIN diode (MACOM MADP-000907–14020) is employed to connect one edge of the patch to the ground through a metal via. Thus an anisotropic unit cell with binary coding reflective performance is obtained along the *x* direction. As shown in Fig. 1(b), to facilitate the biasing in practical implementations, we also introduce a direct-current (DC) circuit in the unit cell topology. The deliberately designed bias circuit includes the quarter-wavelength microstrip line, the open-ended radial stub and DC signal line. The bias point is located at the equivalent zero-electric filed point under x-polarized incidence. The quarter-wavelength microstrip line and the open-ended radial stub are employed to choke the radio frequency (RF) signal and ensure a good isolation between the DC and RF performance^35^. In simulations, the PIN diode is modeled as an equivalent circuit shown in Fig. 1(c,d) when it is switched ON or OFF, respectively. The simulated reflection performances using Ansys HFSS are plotted in Fig. 1(e,f). It is observed that almost total energy is reflected for both *x*- and *y*-polarized incidences. The slightly higher energy loss at ON state under *x*-polarized incidence is resulted from the large equivalent resistance shown in Fig. 1(c). As seen in Fig. 1(f), due to the asymmetrical integration of the PIN diode, distinct reflection phase is obtained for the *x*-polarized incidence when biasing the PIN diode ON or OFF. Moreover, the reflection phase for *y*-polarized incidence is very similar to that for *x*-polarized incidence at ON state. Figure 1(g) further plots the reflection phase differences. Apparently, effective phase difference is observed for *x* polarization with different PIN diode states and for PIN diode working at OFF state with different polarizations. For both cases, a perfect binary coding phase (180° phase difference) is achieved at the design frequency of 11.1 GHz.

Next, the proposed coding unit cell is periodically arranged to construct a programmable metasurface. As an example, a metasurface containing 1600 unit cells is demonstrated. Two types of illuminating sources, namely a plane wave and a point source, are considered. For *x*- or *y*-polarized incidence, the scattering field of the metasurface can be generally expressed as


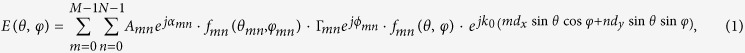


where ***A***_mn_, ***α***_mn_ are the illuminating amplitude and phase, Г_mn_, ***ϕ***_***mn***_ are the reflection amplitude and phase, ***f***_*mn*_(***θ, φ***) is the scattering pattern, ***θ***_***mn***_, ***φ***_***mn***_ are the elevation and azimuth angles of the source relative to the unit cell, *d*_*x*_ and *d*_*y*_ represent the periodicity in *x* and *y* directions. For the proposed microstrip patch unit cell, we assume ***f***_*mn*_(***θ, φ*****)=cos *****θ***, and then Eq. (1) is rewritten as


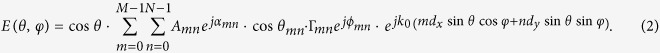


Observing Eq. (2), one can deduce that the scattering field of the metasurface for a specific source can be readily predicted by combing the working states of all unit cells. It is worthwhile to point out that the two possible values of ***ϕ***_***mn***_ can be encoded into a ‘1’ or ‘0’^32^. Then a binary coding matrix corresponding to the whole metasurface can be expressed as


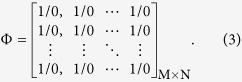


**Figure 2:**
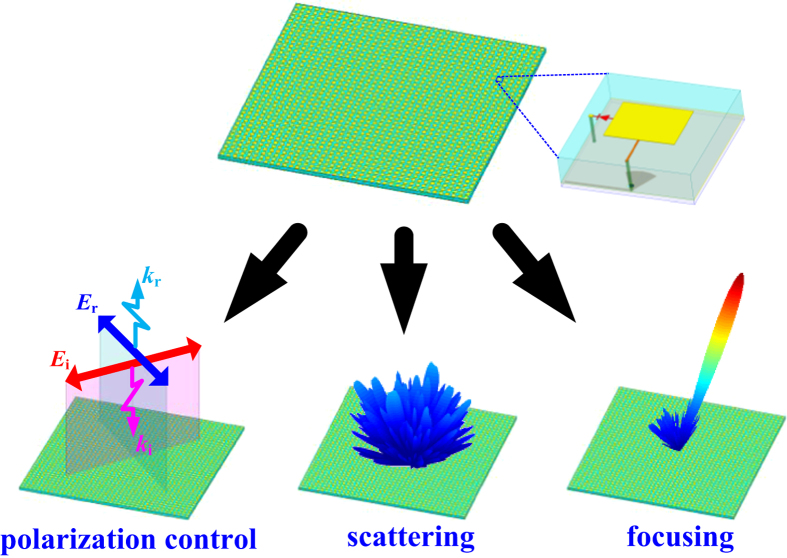
Schematic view of multiple functions for proposed metasurface.

At this point, various functions such as those demonstrated in Fig. 2 can be attained by simply optimizing the binary coding matrix.

To achieve the various functionalities, genetic algorithm (GA)^36^ is adopted to obtain the optimal coding matrix. In GA, the initial population is normally expressed by a group of coding sequences and evaluated simultaneously. Thus it is an effective global optimizer naturally suitable for binary coding and has been widely used in diverse electromagnetic problems. In our simulation, the GA process includes selective reproduction, crossing over, mutation and inversion which is controlled by the fitness and the corresponding probability *Pc, Pm* and *Pi.* Note that the simulation time of a metasurface using Eq. (2) will increase sharply with a large aperture size as a result of the double summation. It makes the GA-based optimization rather inefficient due to the iterative process. To solve this problem, a 2D inverse discrete Fourier transform (IDFT) is introduced and then Eq. (2) is deduced as


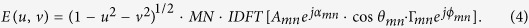


Here, *u* and *v* are defined by


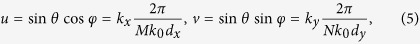


where


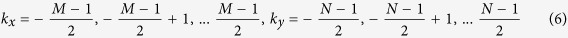


By using the 2D inverse fast Fourier transform (2D-IFFT) technique, the computation cost in one simulation decreases from (MN)^2^ to MN × log(MN) when substituting Eq. (2) with Eq. (4), which is critical for the analysis of a large aperture metasurface. Hence, a much more efficient optimization is achieved.

**Figure 3:**
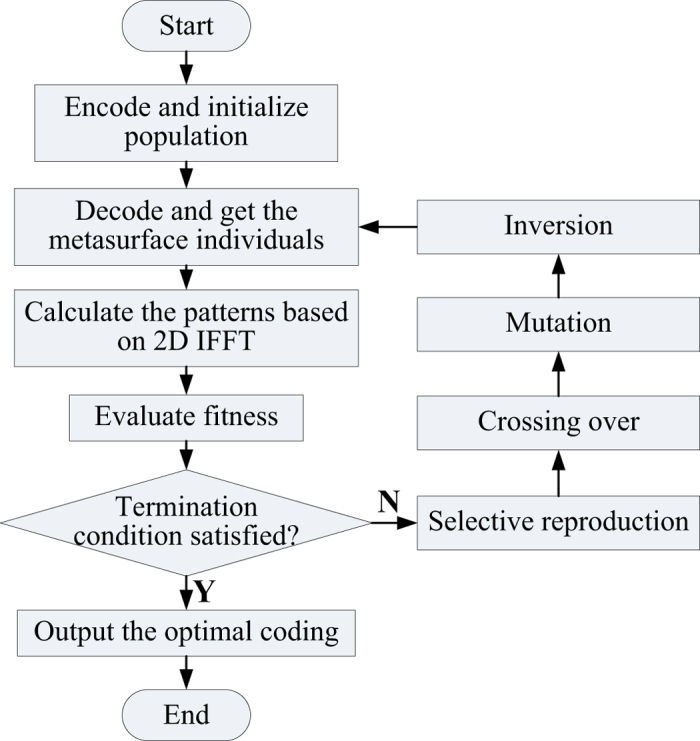
Flowchart of genetic algorithm (GA) based optimization for various functions.

Figure 3 illustrates the flowchart of the GA-based optimization process coupled to the scattering pattern simulations. The initial coding matrices population is decoded into a group of metasurfaces, and the corresponding scattering performances are simulated and evaluated by minimizing a cost function. Then a genetic process is performed to update the individuals until an optimal coding matrix is found. Note that the different functionalities of the metasurface are embodied in the decisive cost function. For example, the cost function for a diffusion metasurface can be expressed as


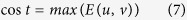


Or for a metasurface with tailored pattern, it can be expressed as


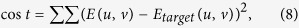


where *E*_target_(*u,v*) is the desired pattern.

### Multiple functionalities of the metasurface

**Figure 4:**
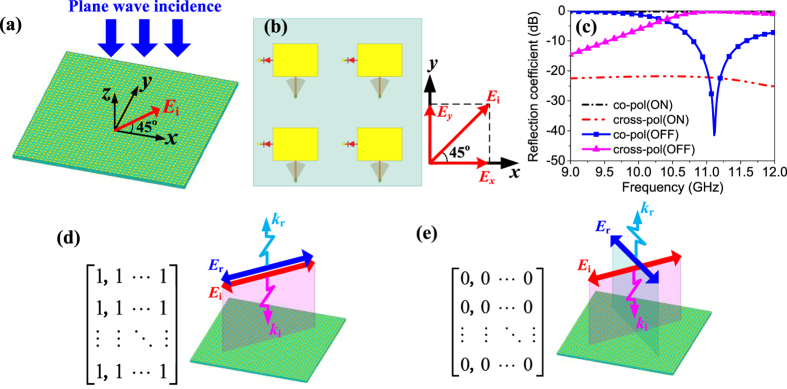
Reconfigurable polarization property. (**a**) The proposed metasurface with normal incident plane electromagnetic wave polarized by 45° with respect to the *x* axis. The metasurface is composed of the proposed unit cells periodically arranged in a square lattice. (**b**) Local zoom of the metasurface and the decomposition of incident electric field. (**c**) Simulated reflection coefficients. (**d**) All ‘1’ coding and sketch map of polarization unaltered reflection. (**e**) All ‘0’ coding and sketch map of polarization rotated reflection.

The functionalities of the metasurface illuminated by a normal incident plane wave are presented first. As discussed previously, the proposed unit cell demonstrates distinct reconfigurable performance for *x*- and *y*- polarized incidences. Exploiting this anisotropic characteristic, a reconfigurable polarization conversion can be obtained when all the unit cells in the metasurface working at ON or OFF state simultaneously, thus resulting in a uniform ‘1’ or ‘0’ coding matrix. To clarify this point, a linearly polarized incident wave with electric field 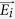 45° off the *x* axis is considered, as shown in Fig. 4(a,b). We decompose 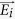 into *x* and *y* components and then the reflected field 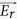 can be expressed as


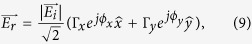


where Г_*x*_ and *ϕ*_*x*_ denote the reflection amplitude and phase along *x* direction, Г_*y*_ and *ϕ*_*y*_ denote the reflection amplitude and phase along *y* direction, 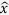 and 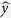 represent the unit vectors in *x* and *y* directions, respectively. Assume Г_*x*=_Г_*y*=_Г, then Eq. (9) can be further rewritten as


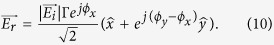


It is deduced from Eq. (10) that a reflection with the same polarization will be obtained when *ϕ*_*y−*_*ϕ*_*x*=*0*_, while an orthogonally polarized reflection will be obtained when *ϕ*_*y−*_*ϕ*_*x*=_*π*, which are verified by the simulated coefficients shown in Fig. 4(c) and visually depicted in Fig. 4(d,e). As can be seen at 11.1 GHz, the parallel reflection is near 0 dB and the orthogonal reflection is below −20 dB for ON state, while the opposite conclusion is observed for OFF state, indicating a reconfigurable polarization function.

**Figure 5:**
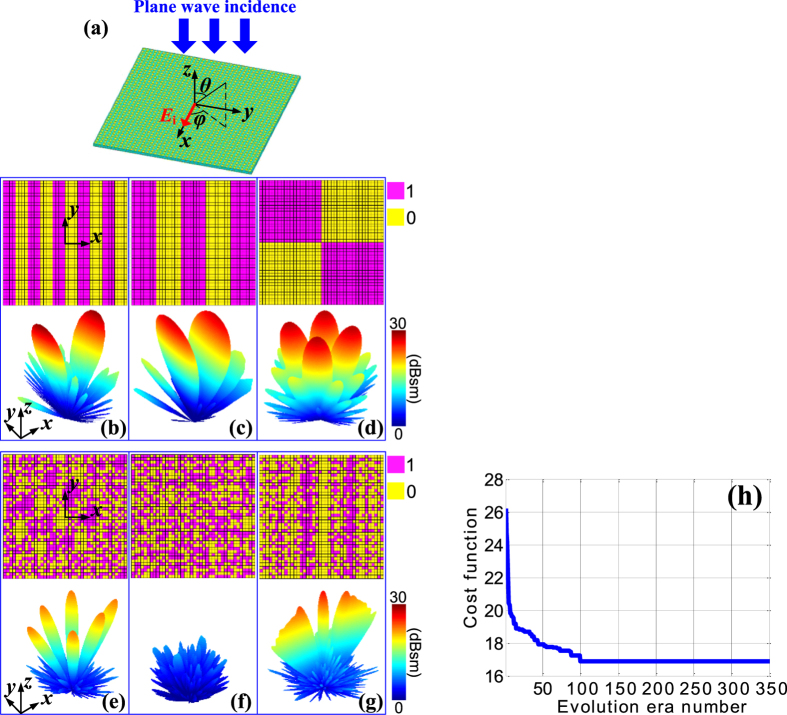
Diversified scattering properties with plane wave incidence. (**a**) The proposed metasurface illuminated by an *x*-polarized plane wave. (**b–d**) Regular binary coding matrices and the corresponding anomalous scattering patterns. (**e–g**) Optimized irregular binary coding matrices and the corresponding scattering patterns. (**h**) Convergence curve of GA-based optimization for (**f**).

Besides the polarization conversion, versatile scattering can also be achieved when the incident plane wave is *x* polarized (seen in Fig. 5(a)). To illustrate this point, anomalous reflections with regular coding matrices are demonstrated first. As displayed in Fig. 5(b,c), by periodically arranging ‘1’ or ‘0’ lattices in alternative columns, abrupt phase variations along *x* direction are generated and the reflection is directed to two off-normal angles, which is verified by the corresponding simulated patterns. Moreover, the derived beam pointings in the two cases, i.e. ( ± 16.2°, 0°) and ( ± 7.9°, 0°), coincide well with the theoretical predictions based on generalized Snell’s law^7^, which are ( ± 16.3°, 0°) and ( ± 8.1°, 0°), respectively. When a chess-board binary coding configuration shown in Fig. 5(d) is applied, the expected reflection split into four lobes in the diagonal directions^37^ is also verified by the simulated result. While the regular coding matrices and the corresponding scattering patterns are successfully demonstrated, it becomes challenging to directly obtain the corresponding coding matrix for an irregular or tailored pattern. In light of this, the GA-based optimization process is utilized. As examples, Fig. 5(e–g) show several optimized coding matrices and the simulated scattering patterns. Apparently, six lobes, diffusion, as well as a shaped pattern are achieved, proving the effectiveness of the optimization method and the diverse scattering functionalities of the proposed metasurface. Furthermore, take the case shown in Fig. 5(f) for example, the optimization convergence curve is plotted in Fig. 5(h). In simulation, the evolution era number is set as 350. The population size of each era is 100. Each individual in the population consists of 1600 binary codes. The initial population is generated randomly in this special case. The probability *Pc*, *Pm* and *Pi* are set as 0.85, 0.15, and 0.2, respectively. The cost function is expressed in Eq. (7). It is observed from Fig. 5(h) that the curve converges quickly and becomes steady after 100 eras, suggesting the high efficiency of GA optimization. It is worthwhile to point out the same optimization has also been performed without using IFFT technique. Although the similar result is obtained, the elapsed time increases from 0.16 hour to 26.83 hours, which verifies the high efficiency of the GA and IFFT combined optimization process.

**Figure 6:**
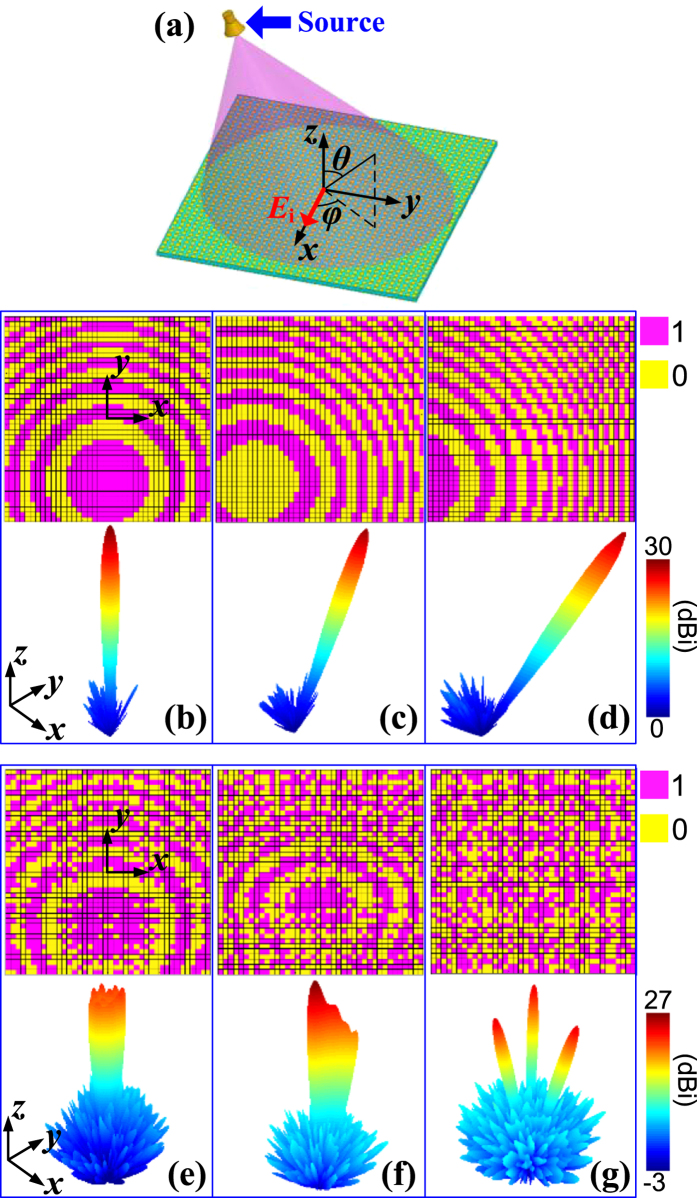
Dynamic focusing properties with a point source. (**a**) The proposed metasurface illuminated by a horn antenna to imitate a point source. (**b–d**) Binary coding matrices and the corresponding steering focused beams pointed at 0°, 20° and 40°, respectively. (**e–g**) Binary coding matrices and the corresponding shaped beams.

When illuminated by an *x*-polarized point source, the metasurface can be regarded as an agile reflective antenna for planar focusing. Here we use a conical horn antenna, as shown in Fig. 6(a), to imitate the point source. In numerical simulations, the horn is located along −*y* axis with a distance of 398 mm above the metasurface and an offset angle of 20° from the *z* axis. According to array theory^38^, a focused beam in a desired direction will be obtained once an equal-phase wavefront is formed. Regarding the reflective antenna, the reflection phase of each unit cell should compensate the spatial phase delay from the point source to the desired beam direction. Since the proposed unit cell only has a binary coding phase, the continuous compensation phase is then discretized into the two quantized values. Thus the theoretical phase distributions are used for single focused beams. As examples, focused beams pointed at (0°, 0°), (20°, 0°) and (40°, 0°) and the corresponding quantized coding matrices are demonstrated in Fig. 6(b–d), respectively. From which, well-defined main beams and relatively low side lobe levels (SLLs) are clearly observed. Take the case presented in Fig. 6(c) for example, the derived beam pointing is (19.9°, 0°), matching well with the desired (20°, 0°) and the SLL is below −22 dB. Moreover, the obtained beams with different pointings also imply that the metasurface is capable of beam steering. Furthermore, various advanced patterns such as shaped beams are also achieved using the efficient GA optimization procedure. For demonstration, three representative cases, i.e. a broad beam, a cosecant shaped beam, a triple-beam, and their corresponding coding matrices, are demonstrated in Fig. 6(e–g), respectively. The excellent beams observed in all cases further verify the powerful focusing capability of the metasurface.

### Fabrication and measurements

**Figure 7:**
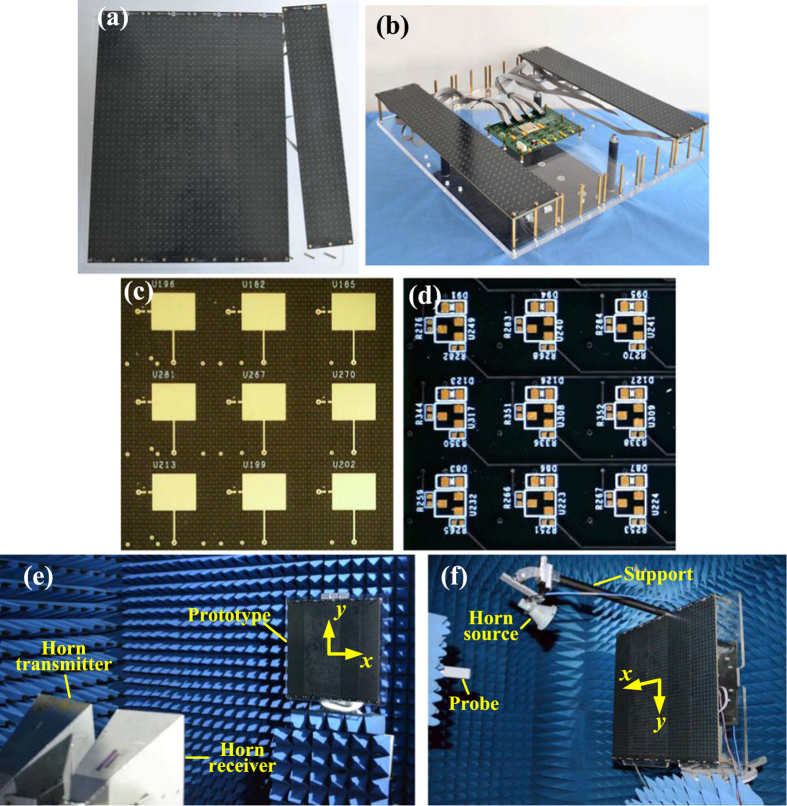
Fabricated prototype and experiments. (**a**) Proposed metasurface constructed by 5 identical sub-metasurfaces. (**b**) FPGA control board and the sub-metasurfaces. (**c**) Local zoom of a sub-metasurface without solder mask and soldering. (**d**) Local zoom of the back of a sub-metasurface. (**e**) Measurement setup for reconfigurable polarization and agile scattering performance. (**f**) Measurement setup for beam-focusing and beam-shaping performance.

In order to validate the simulated results, the proposed metasurface has been fabricated using the standard printed circuit board (PCB) technology. Then electronic devices such as PIN diodes are soldered on the metasurfaces. As displayed in Fig. 7(a), five identical sub-metasurfaces are pieced together to construct the entire prototype. This structure also allows a more flexible aperture size by adding or reducing the sub-metasurfaces as required. To reduce the complexity of the wiring and the control system, a FPGA is employed to distribute the 1600 control data in parallel to 200 shift registers in practical implementations. Each shift register controls 8 PIN diodes sequentially. Thus real-time controlling of each unit cell is realized individually. The similar operating principle has been presented in ref. 35. As seen in Fig. 7(b), the FPGA based control board, placed at the back of the metasurface, is connected with each sub-metasuface through fixable winding wires. Figure 7(c,d) show the local zooms of the front and back of the proposed metasurface, from which the biasing can be observed. Prior to measurements, the pre-designed coding matrices are stored in a computer that is connected to the control board. Hence, the aforementioned various functions can be switched rapidly through a real-time communication of the computer, the control board, and the metasurface.

**Figure 8:**
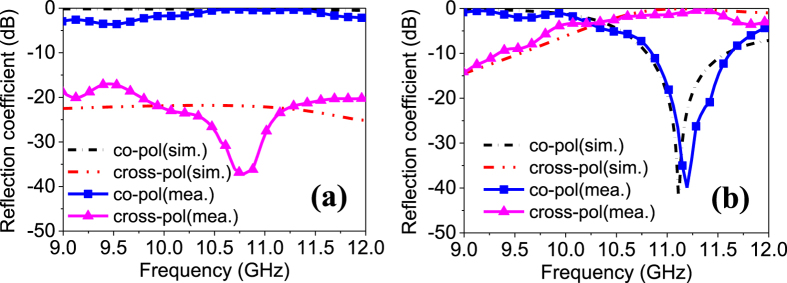
Measurement of reconfigurable polarization conversion. (**a**) Reflection coefficients when all the unit cells work at “1” states (all 1 coding). (**b**) Reflection coefficients when all the unit cells work at “0” states (all 0 coding).

**Figure 9:**
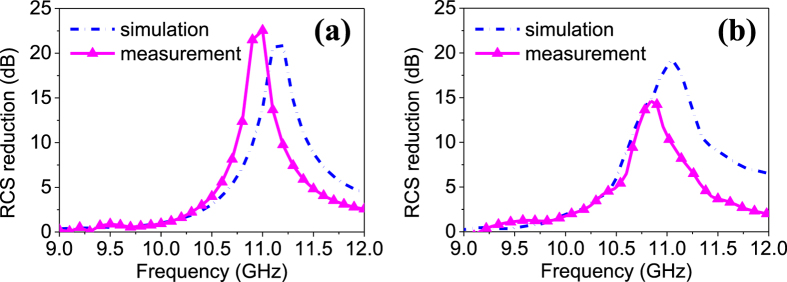
Measurement of RCS reduction versus frequency. The proposed metasurface and a metallic plate with the same size are measured, respectively, and then the RCS reduction is obtained by subtracting the results. (**a**) Measured and simulated RCS reduction with chess-board coding shown in Fig. 5(d). (**b**) Measured and simulated RCS reduction with optimized diffusion coding shown in Fig. 5(f).

For plane wave incidence cases, the metasurface is measured using free-space wave method^39^. As shown in Fig. 7(c), two broadband horns connected to a vector network analyzer (VNA) are used to transmit the EM wave and receive the reflected wave. In this way, the scattering signal can be obtained from the measured S parameters. It is worthwhile to point out that the two horns are set as *x*-polarized that is the same as the metasurface in scattering measurements, while in polarization conversation measurements, the transmitting horn is rotated by 45° with respect to the *x* axis and the receiving horn is rotated by  ± 45° off the *x* axis, respectively. Moreover, a metallic plate with the same aperture size of the metasurface is also measured in all cases for calibration. As shown in Fig. 8, rotated polarization is obtained around 11.2 GHz for a uniform ‘0’ coding metasurface, meanwhile, unaltered polarization is obtained for a uniform ‘1’ coding, proving the reconfigurable polarization conversion performance. Good agreements between simulations and measurements are observed in both figures. The simulations of versatile scattering performance are verified by measuring the radar cross section (RCS) reduction of a regular coding and a GA optimized coding. Due to the facility limitation, only monostatic RCS is tested. The RCS at the normal direction is measured at different frequencies and compared with simulated results. As shown in Fig. 9, significant RCS reduction for chess-board coding and diffusion coding are obtained around 11.0 GHz and 10.8 GHz in measurements, respectively, showing close agreements with the simulations. The peak RCS reduction in Fig. 9(a) is better than that in Fig. 9(b), which is because the chess-board coding dramatically reduces the broadside RCS while the optimized coding is designed to obtain even RCS in the entire space. A slight frequency shift in measurement is mainly attributed to the mutual couplings of adjacent non-uniform unit cell as well as the edge diffraction.

**Figure 10:**
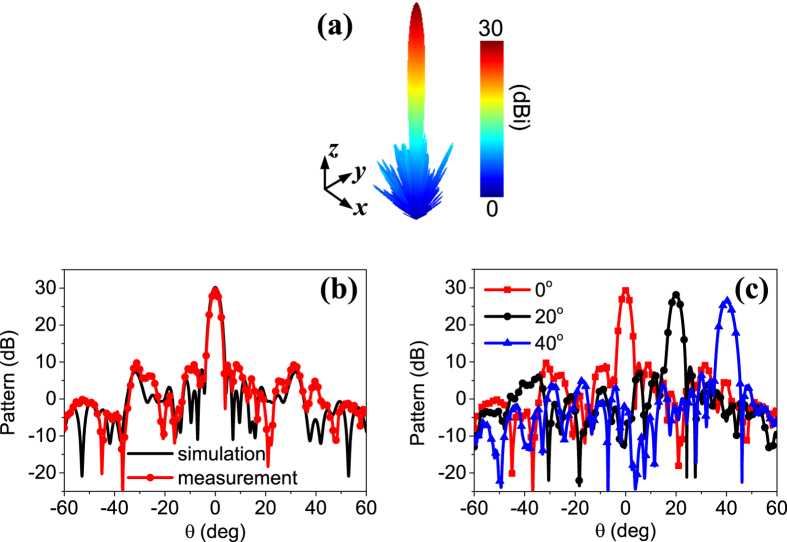
Measurement of beam focusing and beam steering performance. (**a**) Measured 3-dimensional focused beam. (**b**) Measured and simulated focused beams pointed at (0°, 0°). (**c**) Measured steering beams in *xoz* plane. The corresponding coding matrices and the simulated results are presented in Fig. 6(b–d), respectively.

**Figure 11:**
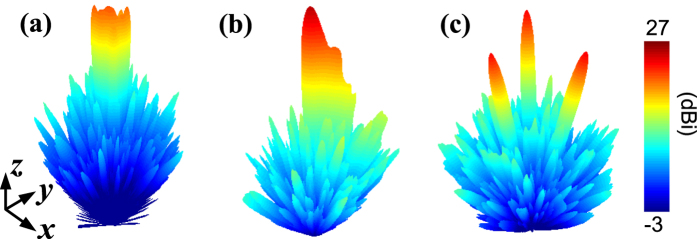
Measurement of beam forming performance. (**a**) Broad beam. (**b**) Cosecant shaped beam. (**c**) Triple-beam. The corresponding coding matrices and the simulated results are presented in Fig. 6(e–g), respectively.

For the point source illumination, a near-field test method^38^ is adopted, as shown in Fig. 7(d). The scattering patterns of the metasurface are obtained through near field to far field transformation. Figure 10 presents the measured focused beams. It shows that the measured results coincide well with the simulations, in terms of both 3-dimensional beams (Figs 10(a) and 6(b)) and the 2-dimensional plots (Fig. 10(b)). For brevity, the measured steering beams corresponding to Fig. 6(b–d) are illustrated in the scanning plane in Fig. 10(c). The well-defined beams further verify that the proposed metasurface not only can collimate the beam, but also can electronically steer the beam. Finally, the broad beam, the cosecant shaped beam and the triple-beam presented in Fig. 6(e–g) are also measured. The close agreements between measurements (Fig. 11(a–c)) and simulations (Fig. 6(e–g)) prove the dynamic beam focusing functionality of the proposed metasurface.

## Conclusion

A programmable metasurface containing 1600 individually-controlled unit cells has been realized to demonstrate the dynamic manipulation of electromagnetic wave. By incorporating one PIN diode into each unit cell, a binary coded phase for a single polarization is obtained. Utilizing this characteristic, reconfigurable polarization conversation is realized. Then a binary coding matrix, genetic algorithm, and inverse fast Fourier transform (IFFT) technique are combined and applied for the relatively large metasurface to obtain various scattering and focusing performance. As examples, anomalous reflection, diffusion, as well as shaped scattering are realized for plane wave incidence. Focused beams, steering beams, and shaped beams are demonstrated for a point source illumination. These functionalities are also confirmed by measuring a practical prototype implemented by 5 identical sub-metasurfaces controlled by a field-programmable gate array (FPGA). The powerful manipulating ability for electromagnetic wave indicates that the proposed metasurface has great promise for future applications.

## Additional Information

**How to cite this article**: Yang, H. *et al*. A programmable metasurface with dynamic polarization, scattering and focusing control. *Sci. Rep.* **6**, 35692; doi: 10.1038/srep35692 (2016).
